# Health system collapse 45 days after the detection of COVID-19 in Ceará, Northeast Brazil: a preliminary analysis

**DOI:** 10.1590/0037-8682-0354-2020

**Published:** 2020-07-03

**Authors:** Daniele Rocha Queiros Lemos, Sarah Mendes D’Angelo, Luis Arthur Brasil Gadelha Farias, Magda Moura Almeida, Ricristhi Gonçalves Gomes, Geovana Praça Pinto, Josafa Nascimento Cavalcante, Levi Ximenes Feijão, Ana Rita Paulo Cardoso, Thaisy Brasil Ricarte Lima, Pâmela Maria Costa Linhares, Liana Perdigão Mello, Tania Mara Coelho, Luciano Pamplona de Góes Cavalcanti

**Affiliations:** 1 Centro Universitário Christus, Faculdade de Medicina, Fortaleza, CE, Brasil.; 2 Secretaria de Saúde do Estado do Ceará, Fortaleza, CE, Brasil.; 3 Universidade Federal do Ceará, Faculdade de Medicina, Fortaleza, CE, Brasil.; 4 Hospital São José de Doenças Infecciosas, Fortaleza, CE, Brasil.; 5 Laboratório Central de Saúde Pública do Ceará, Fortaleza, CE, Brasil.

**Keywords:** COVID-19, Ecological study, Epidemiology, Infectious diseases, Brazil

## Abstract

**INTRODUCTION:**

COVID-19 emerged in late 2019 and quickly became a serious public health problem worldwide. This study aim to describe the epidemiological course of cases and deaths due to COVID-19 and their impact on hospital bed occupancy rates in the first 45 days of the epidemic in the state of Ceará, Northeastern Brazil.

**METHODS:**

The study used an ecological design with data gathered from multiple government and health care sources. Data were analyzed using Epi Info software.

**RESULTS:**

The first cases were confirmed on March 15, 2020. After 45 days, 37,268 cases reported in 85.9% of Ceará’s municipalities, with 1,019 deaths. Laboratory test positivity reached 84.8% at the end of April, a period in which more than 700 daily tests were processed. The average age of cases was 67 (<1 - 101) years, most occurred in a hospital environment (91.9%), and 58% required hospitalization in an ICU bed. The average time between the onset of symptoms and death was 18 (1 - 56) days. Patients who died in the hospital had spent an average of six (0 - 40) days hospitalized. Across Ceará, the bed occupancy rate reached 71.3% in the wards and 80.5% in the ICU.

**CONCLUSIONS:**

The first 45 days of the COVID-19 epidemic in Ceará revealed a large number of cases and deaths, spreading initially among the population with a high socioeconomic status. Despite the efforts by the health services and social isolation measures the health system still collapsed.

## INTRODUCTION

The novel coronavirus SARS-CoV-2, the etiological agent of COVID-19, emerged in Wuhan, China in December 2019 and quickly spread to other countries^1^ ^,^ ^2^. Due to the rapid increase in the number of cases, on March 11, 2020, the World Health Organization (WHO) declared it to be a pandemic^3^. One month after the declaration, more than two million people worldwide had been infected and 135,000 deaths had been registered across 213 countries^4^. Worldwide, health systems faced the need to adapt to a critical overload on services, and a shortage of health care professionals and personal protective equipment^5^ ^,^ ^6^.

In Brazil, the first case of COVID-19 was confirmed on February 26, 2020, and the first death on March 17, both in the state of São Paulo^7^. Community transmission was officially recognized in Brazil on March 20, 2020^8^. Through May 5, 2020, there were more than 110,000 confirmed cases and approximately 8,000 deaths, with a mortality rate of 6.9%. The three most affected states were São Paulo (34,053 deaths), Rio de Janeiro (12,391 deaths), and Ceará (11,470 deaths)^9^.

The state of Ceará in Northeast Brazil was one of the first to confirm sustained transmission. Within 45 days of confirmation of its first case, Ceará had registered the third highest number of deaths in the country. The exponential increase in cases and deaths imposed a series of challenges to meet the demand for care, with a real possibility of a collapse of the health services system. The Brazilian government enacted social isolation regulations on March 19 (Decree 33,519) and a lockdown on May 8 (Decree 33,547). Considerable effort was put into expanding the capacity of emergency services, emergency department care, and laboratory testing, as well as the increasing the number of intensive care (ICU) beds^10^.

We describe the epidemiological scenario of cases and deaths from COVID-19 and their impact on hospital bed occupancy rate in the first 45 days (February 17 to April 27, 2020) of the epidemic in Ceará, Northeastern Brazil. 

## METHODS

### Study type

The study used an ecological design to compare confirmed COVID-19 cases and deaths to bed occupancy rates in Ceará. In addition, we describe the actions implemented during the first 45 days of the epidemic.

### Data sources

Data were collected from six different sources:

1. REDCap - Database in which all suspected and confirmed cases of COVID-19 were recorded from the beginning of the epidemic until April 27, 2020 (45 days after the first known case occurred).
2. SIVEP - Gripe - The National Influenza Epidemiological Surveillance Information System that records all cases of severe respiratory infections and related deaths.
3. e-SUS Notifica - A system developed specifically to meet the high demand for notifications of COVID-19, recording mild and moderate cases of the disease that have undergone laboratory investigation.
4. Ceará state civil registry - The number and cause of verified deaths.
5. Central Laboratory of Public Health of Ceará - Confirmatory laboratory testing results.
6. Unified Health System, Ceará Regulation Center - Hospital admissions.

### Cases Definitions

We followed the case definitions below for suspected cases of COVID-19:

1) An acute respiratory condition characterized by a fever or feverish sensation, even if only reported, accompanied by cough OR sore throat OR runny nose OR breathing difficulty. In the case of children, nasal obstruction was also acceptable in the absence of another specific diagnosis. In the case of the elderly, a reported or diagnosed fever was optional. 2) Specific worsening criteria such as syncope, mental confusion, excessive sleepiness, irritability, and loss of appetite. 3) Dyspnea / respiratory discomfort OR persistent pressure in the chest OR O2 saturation less than 95% in room air OR bluish color of the lips or face. 4) In children, in addition to the previous items, nasal flaring, cyanosis, intercostal circulation, dehydration, or lack of appetite.

We followed the case definitions below for a confirmed case of COVID-19: a suspected case with molecular biology (RT-PCR in real time) detection of the SARS-CoV-2 virus OR a positive immunological test for antibody detection (rapid or classic serology) OR a history of close or home contact with a laboratory-confirmed case for COVID-19 within seven days before the onset of symptoms, and for which it was not possible to perform laboratory testing.

### Study variables and data analysis

The variables used in this study were sex, age group, date of onset of symptoms, whether the subject had been hospitalized, place of hospitalization (public or private), date of hospitalization, the time between first symptoms and hospitalization, whether the patient had been admitted to an intensive care unit, the time between the first symptoms and admission to the intensive care unit, laboratory diagnosis, outcome (discharged with resolved symptoms or death), date and place of death (if occurred), municipality of residence, pre-admission signs and symptoms, the ward occupancy rate, and the number (total and occupied) of ICU beds on the day the patient was admitted. All data were analyzed using Epi Info software version 7.0 (U.S. Centers for Disease Control and Prevention, Atlanta, Georgia).

### Ethical aspects

All ethical principles provided for in the Resolution of the National Health Council (CNS-translated) No. 466, of December 12, 2012, were respected. The study design was approved by the State Secretariat of Health of Ceará.

## RESULTS

**FIGURE 1:**
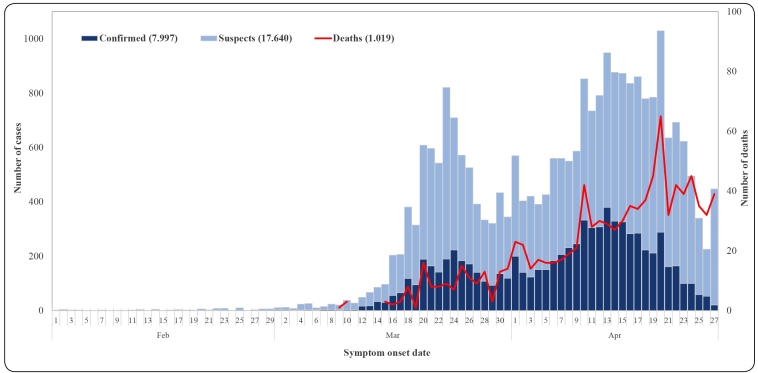
Number and temporal distribution of COVID-19 cases, by epidemiological week of symptom onset. Ceará, Brazil, 2020.

**FIGURE 2:**
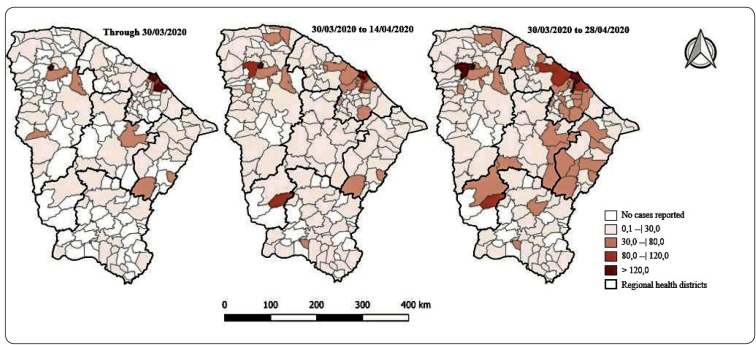
Spatial distribution of municipalities with confirmed COVID-19 cases in the first 45 days of the epidemic, by epidemiological week. Ceará, Brazil, 2020.

The first confirmed cases of COVID-19 in Ceará were diagnosed on March 15, 2020, with onsets of symptoms on the 10th (two cases) and the 11th (one case). Within 45 days of the country’s first known case, 37,268 cases had been confirmed in 85.9% of Brazil’s 184 municipalities (Figures 1 and Figure 2). Of the confirmed cases, 7,833 (21.0%) were laboratory-confirmed, another 20,791 were under investigation and 1,019 were confirmed COVID-19-related deaths. Epidemic week 20 had the highest number of reported cases and the peak of deaths.

The distribution of cases was initially the most widespread in Fortaleza, Ceará’s capital city, later overtaken by metropolitan municipalities in which the highest incidences were identified (over 120 cases per 100,000 inhabitants). The virus also spread through municipalities in the Northern region, which also had incidences above 120 cases per 100,000 inhabitants.

**FIGURE 3:**
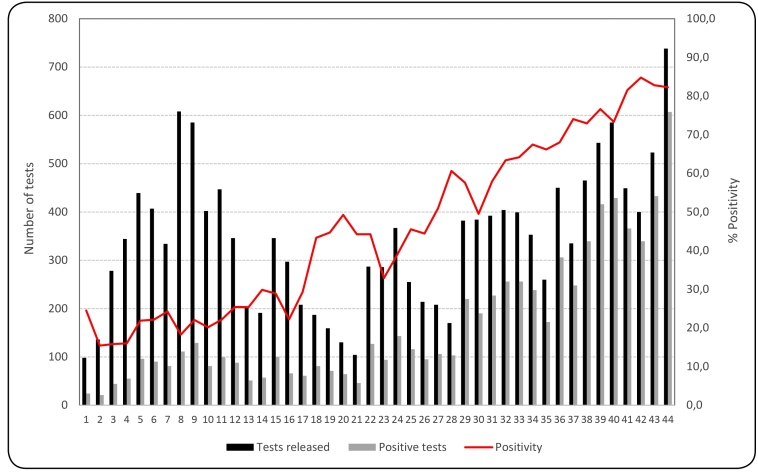
Number of tests and positivity of COVID-19 tests performed during the first 45 days of the epidemic. Ceará, Brazil, 2020.

During the 45-day period, the Central Laboratory of Public Health of Ceará (LACEN-CE) processed more than 15,000 molecular biology exams, reaching 738 tests in a single day (April 27). The lowest positive rate among the examinations was registered on March 21 (15.4%) and the highest on April 26 (84.8%). In the first 15 days, the average positivity was 21.9%, increasing to 46.2% and 73.6% over the next 15 and 30 days, respectively (Figure 3).

The first death in Ceará was confirmed on March 23; within four days another 10 had been confirmed. Ten days after that, 57 more deaths had been confirmed. Subsequently, the number of confirmed deaths doubled approximately every seven days, reaching the highest single day occurrence on May 1st (53 deaths). 

Females made up a higher percentage than males of reported and confirmed cases (54.7%) but deaths occurred in a greater proportion in males (58.1%) (Table 1). The same pattern occurred in age groups where a predominance of cases were in people aged 20 to 59 years (70.3%), but the percentage of deaths was highest for those over 70 years of age (52.9%).

**TABLE 1:** Demographic and medical characteristics of COVID-19 cases and deaths in the first 45 days of the epidemic. Ceará, Brazil, 2020.

Variables	Notified		Confirmed		Deaths	
	N	%	N	%	N	%
**Gender**						
Male	16 874	45.3	3 666	46.8	592	58.1
Female	20 332	54.7	4 167	53.2	427	41.9
**Age group**						
< 1 year	570	1.5	67	0.9	0	0.0
1- 9 years	1 274	3.4	93	1.2	3	0.3
10- 19 years	1 097	2.9	134	1,7	2	0.2
20-59 years	26 031	70.3	5 492	70.1	287	28.2
60-69 years	3 727	10.1	945	12.1	188	18.4
> 70 years	4 313	11.7	1 091	13.9	539	52.9
**Needed hospitalization**						
Yes	2 647	7.1	1 288	16.4	878	91.9
No	34 621	92.9	6 545	83.5	77	8.1
**Hospitalization in the Intensive Care Unit (ICU)**						
Yes	777	2,1	499	6.4	473	58.0
No	36 491	97.9	7 334	93.6	342	42.0

The average age of the cases that progressed to death was 67 (1-101) years old, with more than half (52.9%) occurring in people over 69 years old (Table 1). The main symptoms reported among these cases were dyspnea (86.0%), fever (85.2%), cough (84.7%), respiratory distress (77.1%), sore throat (21.5%), diarrhea (14.1%), and vomiting (7.5%). The most common comorbidities were heart disease (66.5%) and diabetes (58.3%). Most deaths occurred in a hospital setting (91.9%), more than one-half required ICU bed hospitalization (58.0%), and 480 (53.9%) needed ventilator support. The average time between the onset of symptoms and death was 18 (1-56) days. The cases that evolved to death took 6 (0 - 40) days to be hospitalized and among those who were hospitalized, the average time in bed was 8 (1 - 49) days Considering the public hospital network in Ceará, on the 45th day of the epidemic COVID-19 patients occupied 655 ward beds, 421 ICU beds, and 376 beds with respirators. This represented 71.3% (23.8%-100.0%) of all ward beds, 80.5% (40.0%-100.0%) of all ICU beds, and 74.9% (40.0%-100.0%) of beds with mechanical ventilation (Table 2).

**TABLE 2:** Evaluation of bed use 45 days after the first confirmed case of COVID-19 in Ceará, in 2020.

Health Unit	Infirmary			ICU			Mechanical Ventilation		
	N	Occupied	%*	N	Occupied	%	N	Occupied	%
Hospital Leonardo da Vinci	66	61	92.4	115	83	72.2	74	66	89.2
Hospital Geral de Fortaleza	111	76	68.5	51	46	90.2	49	49	100.0
Hospital Geral Dr César Cals	24	22	91.7	10	10	100.0	10	9	90.0
Hospital São José	82	61	74.4	8	8	100.0	27	22	81.5
Hospital de Messejana	56	56	100.0	59	57	96.6	57	45	78.9
Hospital Infantil Albert Sabin	42	31	73.8	8	8	100.0	8	7	87.5
Hospital Abelardo Gadelha (Caucaia)	26	21	80.8	12	12	100.0	12	10	83.3
Hospital MM (Maracanaú)	13	9	69.2	5	2	40.0	5	2	40.0
Hospital São Vicente de Paula (Itapipoca)	11	9	81.8	10	10	100.0	8	8	100.0
Hospital Batista	124	88	71.0	7	3	42.9	3	2	66.7
Hospital Regional Norte (Sobral)	25	24	96.0	36	25	69.4	23	15	65.2
Hospital Regional SC (Quixeramobim)	33	17	51.5	30	27	90.0	30	19	63.3
Hospital Regional do Cariri (Juazeiro)	21	5	23.8	35	22	62.9	35	18	51.4
Hospital São Vicente (Iguatu)	21	5	23.8	35	22	62.9	35	18	51.4
**Total**	**655**	**485**	**74.0**	**421**	**335**	**79.6**	**376**	**290**	**77.1**

*Percent occupied.

## DISCUSSION

The first 45 days of the COVID-19 epidemic in Ceará showed an explosion in the number of cases and deaths, reaching more than 1,000 confirmed deaths. In the first days after the detection of the first cases, infections spread most rapidly in the city of Fortaleza and municipalities with higher HDI, mainly among users of the supplementary health network. In the following month, infections began to spread to the interior of the state, reaching the periphery of large cities and the most vulnerable social and economic populations. These populations have a higher prevalence of comorbidities, and often have living situations that make effective social isolation unfeasible^11^. This likely contributed to the early exponential increase in the number of cases and deaths.

The symptoms described in this study were similar to those reported for the pandemic for other countries^12^. Our findings show a predominance of male deaths, as was also reported in China^13^. A recent survey of more than 2,000 people in Ceará showed that a significant percentage of females perceive themselves to be at risk of COVID-19, while males reported greater difficulty adhering to social isolation practices^14^. These observations could, in part, explain our findings.

At the beginning of the epidemic, due to concerns about the simultaneous increase of seasonal influenza and the possibility of a new dengue epidemic, the Ceará health department advised that medical care should only be sought for severe symptoms such as shortness of breath, difficulty breathing, or cognitive impairment^15^ ^,^ ^16^. This initial guidance, together with fears in the population of acquiring infection from attending a health facility, probably contributed to the increase in the number of home deaths. By the beginning of April, there were widespread notices by the Ceará health department that everyone, especially those with known risk factors, should seek basic care at the first sign of symptoms. This change reduced the number of home deaths, but also generated a large demand for basic health centers, contributing to the collapse of the outpatient care network.

This study had several limitations. We relied on secondary databases from local surveillance systems. Further, not all cases with laboratory confirmation were by RT-PCR, but those limitations don’t invalidate the results.

The Brazilian Ministry of Health published Decree 10.211 at the end of January, reactivating the Interministerial Executive Group on Public Health Emergency of National and International Importance (GEI-ESPII). Among other duties, the GEI-ESPII is responsible for implementing preparation measures and the official health surveillance response to the COVID-19 epidemic^17^ ^,^ ^18^. It is noteworthy that despite extensive efforts by health services, social isolation measures were not effective in reducing the speed of disease spread to the pace at which the health care network was expanding. This imbalance led to the collapse of the health system^19^ ^,^ ^20^. 

Development and implementation of effective health service responses to COVID-19 has been challenging, especially in poor countries^21^. In this scenario, it is essential to identify effective drugs for early stages of the disease and to develop an efficient vaccine^22^.
